# Commentary: Peripartum Cardiomyopathy in Intensive Care Unit: An Update

**DOI:** 10.3389/fmed.2016.00008

**Published:** 2016-02-19

**Authors:** Murat Biteker, Kadir Uğur Mert, Gurbet Özge Mert

**Affiliations:** ^1^Department of Cardiology, Faculty of Medicine, Muğla University, Muğla, Turkey; ^2^Department of Cardiology, Eskişehir Yunus Emre State Hospital, Eskişehir, Turkey

**Keywords:** cardiomyopathy, peripartum cardiomyopathy, heart failure, systolic, idiopathic cardiomyopathy, pregnancy

We read with interest the article recently published by Dinic et al. entitled “Peripartum Cardiomyopathy in Intensive Care Unit: An Update” (1). However, we have some concerns about the article.

Our first concern is about the definition of peripartum cardiomyopathy (PPCM). The authors defined PPCM as the development of heart failure during the last month of pregnancy or within 5 months after delivery. However, the restriction of the time frame to last month of pregnancy or first 5 months postpartum for diagnosis has been challenged (2). In a study by Elkayam et al., almost 20% of the patients developed symptoms of heart failure and were diagnosed with PPCM earlier than the last gestational month (3). A comparison between patients with early presentation and those with traditional criteria of PPCM revealed no significant differences in age, ethnic background, obstetrical history, and rate of gestational hypertension. Maternal outcome, left ventricular function at the time of diagnosis, and its recovery over time were also similar between the two groups. These findings indicate that PPCM and pregnancy-associated cardiomyopathy are part of the same clinical spectrum, and some patients may present with PPCM symptoms earlier than the last gestational month. Hence, a position statement from a European Society of Cardiology working group on PPCM has eliminated the strict time limit to the diagnosis and expanded the definition of PPCM to “an idiopathic cardiomyopathy presenting with heart failure secondary to left ventricle systolic dysfunction *toward the end of pregnancy* or in the months following delivery, where no other cause of heart failure is found” (4). We think that PPCM is a diagnosis of exclusion and the time frame and echocardiographic cut-offs are arbitrary and can lead to underdiagnosis of PPCM.

Our second concern is about the duration of therapy in patients with PPCM. Recently, we have published results of 42 prospectively followed PPCM patients (5). Twenty patients (47.6%) recovered completely, 10 died (23.8%), and 12 (28.6%) had persistent left ventricular dysfunction. Average time to complete recovery was 19.3 months after initial diagnosis (3–42 months). Four patients (two patients with complete recovery and two patients with partial recovery) showed delayed deterioration (12, 24, 26, and 34 months after diagnosis) during the study period. Of the four patients with spontaneous deterioration of left ventricular function, two patients with partial recovery were receiving full-dosage heart failure treatment, but two patients with complete recovery stopped taking their medications after recovery, and had delayed deterioration at 24 and 34 months. The findings of late deterioration are very important and indicate the need for a close follow-up with periodic determination of cardiac function in women in whom medications are discontinued after complete recovery. Due to probability of either delayed recovery or deterioration of left ventricular function in PPCM, long-term follow-up may be needed not only in non-recovered patients but also in patients with complete recovery.

Our last concern is about the use of levosimendan in PPCM. Although authors referred two case reports, they have omitted to mention the only randomized clinical trial evaluating the role of levosimendan in patients with PPCM. In a prospectively randomized study, we showed that the addition of levosimendan to conventional therapy did not improve the outcome in patients with PPCM (6).

## Author Contributions

KM: review and study design, GM: literature, data collection, and analysis. MB: interpretation and writing.

## Conflict of Interest Statement

There are no relationships with industry posing a conflict of interest in this paper.
